# “Go home and practice”: how shaping feedback to students can foster independent musicianship

**DOI:** 10.3389/fpsyg.2025.1705295

**Published:** 2025-10-28

**Authors:** Camilla dos Santos Silva, Jennifer Blackwell, Gary E. McPherson, Evely Boruchovitch

**Affiliations:** ^1^Universidade Estadual de Campinas, Campinas, Brazil; ^2^Northwestern University Henry and Leigh Bienen School of Music, Evanston, IL, United States; ^3^The University of Melbourne, Melbourne, VIC, Australia

**Keywords:** feedback, visible learning, metacognition, music teacher education, studio teaching, music learning

## Introduction

Students typically receive one instrumental lesson per week, and their learning relies heavily on how they absorb and apply information in their daily practice. Given the essential role of practice, how can teachers ensure the effectiveness of their students' practice between lessons? A substantial body of literature in educational psychology has provided valuable insights into the cognitive and metacognitive dimensions of music practice, with a particular emphasis on learning strategies (e.g., How et al., 2022; McPherson and Zimmerman, 2011). Yet, even after more than two decades of research on the metacognitive aspects of music learning, practice, and performance, challenges remain evident, impacting both beginners (Miksza, 2012; Prichard, 2017, 2021) and advanced musicians (Dos Santos Silva and Marinho, 2025; McPherson et al., 2019; Miksza et al., 2018). The psychological dimensions of self-regulated learning provide a framework for examining how different elements can contribute to efficient music practice (McPherson and Zimmerman, 2011). Motivation, Method, Behavior, Time, Physical Environment, and Social Factors are dimensions that encompass various SRL processes, such as goal setting, self-monitoring, managing practice time and the physical environment, selecting and adapting metacognitive strategies, and seeking help when needed (Zimmerman and Risemberg, 1997).

Complementing research that examines which processes and behaviors should be fostered in music learners, recent work by McPherson and his colleagues has also emphasized the need to improve how these processes are communicated to and understood by students (McPherson and Hattie, 2022; McPherson et al., 2022). Thus, another way to conceptualize how music learners acquire the skills necessary to develop musical and technical skills is by exploring how information provided by the teacher is received and processed by students. Given that the goal of self-regulated learning is student autonomy, providing actionable feedback that translates abstract ideas into learning is at the heart of developing musicianship (Hattie and Timperley, 2007). Students are expected to use the feedback received from their teachers to improve their performance skills, learn how to monitor their practice, and self-evaluate their achievements.

## Conceptualizing feedback

Work by (McPherson et al. 2022) and (Blackwell et al. 2023) demonstrates that feedback is a term widely used in the literature on both music and educational psychology, yet it lacks a strong theoretical conception and definition. Recent research has shown that not all feedback is equally effective, and it is crucial to understand what constitutes effective feedback and how it can be utilized to enhance student learning (McPherson et al., 2022). In this context, Hattie and colleagues have proposed a conceptual framework for effective feedback processes into three different types: Feed Back (How am I going?) refers to assessing students' performance in comparison to criteria such as previously set goals and outcomes, including previous performances, exams, or lessons (McPherson et al., 2022). Feed Up (Where am I going?) provides information that emphasizes learning or performance goals, guiding the student on what can be done in the present to achieve desired outcomes. Feed Forward (Where to next?) is considered the most critical type of feedback (Hattie and Clarke, 2019; McPherson et al., 2022) and refers to communicating to the student the next steps they must take to achieve their goals (Brooks et al., 2019; Hattie, 2011; Hattie and Timperley, 2007; Hattie and Clarke, 2019).

Each feedback type can also connect to four feedback levels: Task level feedback refers to information about the task itself, the outcomes obtained, and ways to achieve better results. Process level feedback addresses how students can improve their effort to perform the task more effectively. Self-regulation level feedback involves modeling metacognitive processes that help learners plan, monitor, and control their behavior as they approach the task. Finally, self level feedback consists of personal comments directed at the student, a type of feedback that is usually regarded as unhelpful and, in some cases, even detrimental to learning (Hattie and Timperley, 2007; Hattie, 2011; Brooks et al., 2019).

The taxonomy of types and levels of feedback articulated by Hattie and colleagues enables a multidimensional perspective when considering the feedback content, temporal organization (past—Feed Back, present—Feed Up, future—Feed Forward), and the resources needed to ensure that feedback is actionable for students, thus allowing learning to be more effective. Feedback that emphasizes self-regulation, learning processes, and how students can learn in the future (Feed-Forward) is essential for developing student self-regulation.

As suggested by the systematic review of (Blackwell et al. 2023), there is a need to develop structured methods for investigating feedback, grounded in established theoretical bases, that can provide a framework for meaningful discussion about effective feedback for music performance learning.

## Original research

(McPherson and Blackwell 2024) drew on Hattie's framework and the method employed by (Brooks et al. 2019) to analyze the occurrence of feedback during 18 university-level instrumental lessons taught by six renowned music teachers. Data were collected through video recordings of these lessons, which were later transcribed and coded according to the types of feedback (Feed Back, Feed Up, or Feed Forward), as well as across the feedback levels (Task, Process, Self-regulation, and Self).

Results revealed that most comments were categorized as Feed Back (83.3%), followed by Feed Forward (16.3%), and there were very few instances of Feed Up (0.4%). Regarding feedback levels, 85.3% of the comments were identified as Task Level, 9.2% as Process Level, and 5.5% as Self-Regulation Level. Notably, these results suggest that very little feedback was directed toward music practice or the development of self-regulation, suggesting that lessons may not be developing essential knowledge and skills for effective practice.

This work yielded valuable insights into how feedback occurs in collegiate music lessons, particularly when compared to results in other fields. (Brooks et al. 2019) found a similar trend in their study in the general education context, but with less discrepancy between types of feedback (for example, they categorized 42% of the comments as feed-back task, while 77% occurred in McPherson and Blackwell's). The authors highlight the importance of developing feedback literacy in both students and teachers, aiming to promote a proactive learning environment.

Following the suggestions proposed by (McPherson and Blackwell 2024) and (Blackwell and Matherne 2024) investigated how preservice music teachers developed their understanding of feedback and applied it by teaching instrument lessons to their peers. During one semester, 11 music education undergraduate students received feedback instruction based on Hattie's Visible Learning theory, in conjunction with their woodwind techniques course. Data were collected through interviews, survey responses, and the researchers' field diary, and coded by the researchers. Data were organized in themes, such as developing understanding of feedback (how they articulated concepts of types and levels of feedback), rapport (providing honest feedback without sounding overly critical), expertise and trust (how feedback is received based on the expertise of the source), and finally, change over time (how participants sought providing feedback that was understood by their peers and more goal-oriented).

## Discussion

These two studies provide complementary evidence about the use of feedback in music performance contexts. (McPherson and Blackwell 2024) focus on identifying the types and levels of feedback, while (Blackwell and Matherne 2024) provide evidence for the need for training in feedback literacy. However, the samples used in these studies differ; McPherson and Blackwell examined feedback practices among renowned studio music teachers, whereas Blackwell and Matherne offered their intervention to music education students teaching a secondary instrument. Furthermore, Blackwell and Matherne retrieved data through self-report instruments and observations, while McPherson and Blackwell used video recordings and a systematic coding of feedback frequency.

The intervention by Blackwell and Matherne suggested that there were changes in how participants articulated concepts of feedback. Initially, they limited themselves to listing the ideas presented in class, but later, they were able to reflect on what makes feedback understandable and actionable. Students were also concerned about maintaining good relationships with their peers so that they could provide honest feedback without sounding overly critical. Regarding how participants receive feedback depending on how much they see the feedback source as an expert, their reflections led them to seek guidance on how to deliver feedback more effectively. These results shed light on ways of increasing the frequency of Feed-Forward at the self-regulation level, which have been identified as the most effective form of feedback in previous literature (Hattie and Timperley, 2007; Wisniewski et al., 2020).

Understanding feedback theory is essential for teachers, as it not only reinforces the characteristics of effective feedback but also helps organize learning resources that promote metacognitive skills in music lessons. When focused on self-regulation, this feedback approach enables teachers to clearly define performance goals, provide guidance on problem-solving, facilitate strategy evaluation and behavior adaptation, and demonstrate to students how to monitor their own practice and assess their performance. Such instruction also seeks to avoid vague practice directions that could hinder musical growth, especially in beginners.

## Educational implications and suggestions for future research

Combining the recommendations from both articles that we have analyzed, we emphasize the need for studies that integrate the identification and categorization of different types and levels of feedback in music lessons with the implementation of interventions aimed at enhancing the understanding of feedback in music lessons.

To complement qualitative research, it would be valuable to gather data that allows for the investigation of large sample sizes and enables some degree of generalization regarding the frequency with which different types and levels of feedback occur in music lessons across various contexts worldwide.

Therefore, future studies should aim to develop and validate a scale that measures the extent to which different types and levels of feedback occur in music lessons across larger and more diverse populations. Additionally, it would be beneficial to statistically test the factors of this scale in relation to the taxonomy proposed by (Hattie and Timperley 2007).

As noted by (McPherson and Blackwell 2024), adapting the Visible Learning Theory to the context of music education presents a significant challenge, particularly because instrumental lessons involve a substantial amount of non-verbal communication. Gesture plays a crucial role in musical discourse, and it is especially important in instrumental instruction, where teachers use gestures to guide students as they play. Future studies could explore gesture as a form of feedback, investigating whether it represents a distinct category or can be integrated into the classifications established by (Hattie and Timperley 2007).

Music performance teachers should receive instruction on how to provide feedback at the self-regulation level, which includes information about how the student can regulate their thoughts, behaviors, and emotions toward their goal, such as preparing for a recital. This level involves a metacognitive approach to feedback (Hattie and Timperley, 2007) and requires self-monitoring and self-recording information during daily practice and music lessons. By doing this, students are able to evaluate and adapt their practice according to the set goals. While feedback at the self-regulation level occurs more commonly when students become independent, teachers can stimulate this approach by modeling self-evaluative and self-monitoring strategies (Matherne and Blackwell, 2023; Brooks et al., 2019).

Music teachers face a challenging role to ensure the link between modeling practice strategies and providing effective feedback. This connection can either facilitate or hinder a student's ability to employ these strategies, regulate their practice, and achieve musical growth.
